# A Novel Sequence in AP180 and CALM Promotes Efficient Clathrin Binding and Assembly

**DOI:** 10.1371/journal.pone.0162050

**Published:** 2016-08-30

**Authors:** Lia Moshkanbaryans, Jing Xue, Jesse Ray Wark, Phillip James Robinson, Mark Evan Graham

**Affiliations:** 1 Synapse Proteomics Group, Children’s Medical Research Institute, The University of Sydney, Westmead, NSW 2145, Australia; 2 Cell Signalling Unit, Children’s Medical Research Institute, The University of Sydney, Westmead, NSW 2145, Australia; University of Edinburgh, UNITED KINGDOM

## Abstract

The clathrin heavy chain N-terminal domain interacts with endocytic adapter proteins via clathrin binding motifs to assemble clathrin triskelia into cages. However, the precise mechanism of clathrin assembly is not yet known. Clathrin assembly protein AP180 has more clathrin binding motifs than any other endocytic protein and has a major role in the assembly of the clathrin coat during synaptic vesicle biogenesis. We now demonstrate that some of the previously identified binding motifs in AP180 may be non-functional and that a non-conventional clathrin binding sequence has a major influence on AP180 function. The related protein, clathrin assembly lymphoid myeloid leukemia protein (CALM), has fewer clathrin binding motifs and functions ubiquitously in clathrin-mediated endocytosis. The C-terminal ~16 kDa sub-domain in AP180, which has relatively high similarity with CALM, was shown in earlier work to have an unexplained role in clathrin binding. We identified the specific sequences in this sub-domain that bind to clathrin. Evidence for a role for these sequences in promoting clathrin binding was examined using *in vitro* and *ex vivo* experiments that compared the clathrin binding ability of site mutants with the wild type sequence. A sequence conserved in both AP180 and CALM (LDSSLA[S/N]LVGNLGI) was found to be the major interaction site and mutation caused a deficit in clathrin assembly, which is the first example of a mutation having this effect. In contrast, single or double mutation of DL(L/F) motifs in full length AP180 had no significant effect on clathrin binding, despite higher clathrin affinity for isolated peptides containing these motifs. We conclude that the novel clathrin interaction sites identified here in CALM and AP180 have a major role in how these proteins interface with clathrin. This work advances the case that AP180 and CALM are required to use a combination of standard clathrin N-terminal domain binding motifs and the sequence identified here for optimal binding and assembling clathrin.

## Introduction

An early event in clathrin-mediated endocytosis (CME) is the formation of a clathrin-coated pit at the plasma membrane which requires clathrin binding to endocytic adaptor proteins [1,2]. Clathrin has a major partnership with assembly protein 180 kDa (AP180) in synaptic vesicle endocytosis (SVE) and with clathrin assembly lymphoid myeloid leukemia protein (CALM) in the ubiquitous process of CME [3]. There are a multitude of adaptor, accessory and uncoating proteins that interact with the clathrin heavy chain N-terminal domain (NTD) throughout various stages of CME. All of these interactions are thought to involve clathrin binding motifs (CBMs), which target multiple NTD binding sites [4–8].

AP180 efficiently assembles clathrin and this has been attributed to its multiple CBMs [7,9]. The D(L/I)(L/F) CBM, which can be considered a degenerate version of the classical clathrin box motif pLΦpΦp (p = polar and Φ = hydrophobic) [4], occurs eleven times in AP180 [7]. The ability of AP180 to assemble clathrin *in vitro* was shown to be directly proportional to the number of CBMs [7]. The NTD has three protein binding sites which can accommodate CBMs [5,8] and AP180 binds clathrin heavy chain in a ratio of 1:3 [3,10]. Thus, the multiple CBMs in AP180 appear to provide the structural basis for assembly of a clathrin cage during SVE [7].

The C-terminal assembly domain (AD) of AP180 assembles clathrin *in vitro* as well as the full length protein [7]. All of the CBMs in AP180 occur in a central clathrin and adaptor protein binding (CLAP) sub-domain (of the AD). The remainder of the assembly domain, the ADΔCLAP, is approximately 16 kDa. The ADΔCLAP binds clathrin cages [11] and extracts clathrin from rat brain lysate [12]. Truncation of parts of the ADΔCLAP renders the remaining AP180 sequence unable to efficiently extract clathrin from rat brain lysate, despite an intact CLAP domain. Furthermore, the dominant negative effect of full length AP180 in a transferrin (Tfn) uptake assay was significantly reduced by C-terminal truncation of 100 amino acids [12]. These observations indicate that one or more sequences in the ADΔCLAP contribute to the AP180-clathrin interaction.

A comparison of AP180 and CALM is important because the ADΔCLAP appears to be a conserved sub-domain [3]. CALM has only one known CBM [13], which is insufficient for CALM to cross-link multiple clathrin NTD. A previous study confirmed at least two *in vitro* clathrin binding sites in human CALM 414–652 [14]. Another study showed that CALM is no longer dominant negative for Tfn uptake following C-terminal truncation [15]. These studies indicate that there are unidentified clathrin binding sequences in the CALM ADΔCLAP.

We tested whether clathrin bound directly to specific sequences in the AP180 and CALM ADΔCLAP. We identified both conserved and non-conserved *in vitro* clathrin binding sequences. We also examined the effect of mutation of these sites on clathrin assembly activity and Tfn uptake. These latter experiments confirmed that the conserved sequence had a function in clathrin binding and assembly.

## Materials and Methods

Buffer reagents were from Sigma-Aldrich and gel electrophoresis reagents were from Bio-Rad. Nitrocellulose membranes and enhanced chemiluminescence reagent were from Perkin-Elmer and Pierce, respectively. Anti-Clathrin [X22], anti-VAMP2 and anti-AP180 were from Abcam. Anti-CALM and anti-β-Actin was from Sigma. Anti-GFP was from Roche. All horseradish peroxidase-conjugated secondary antibodies were from DAKO. Dulbecco’s Modified Eagle’s Medium (DMEM), fetal bovine serum (FBS), phosphate buffered saline (PBS), Trypsin-EDTA and Alexa Fluor 594 labelled transferrin were from Invitrogen. Paraformaldehyde was from Scharlau Chemie. All reagents were of analytical grade unless specified otherwise.

### Peptide array overlay assay

Arrays of 15mer peptides were synthesised directly on cellulose membrane. The PepSpots arrays were synthesised by JPT Technologies. The N-termini of the peptides were acetylated to mimic an internal sequence. Each 15mer is separated from the membrane by an Ala linker at the C-terminus of the 15mer sequence. The 15mer peptides from the ADΔCLAP of AP180 and CALM were designed to ensure an overlap of 12 out of 15 amino acid residues for adjacent peptides on the array, which enables a minimum 3 or more amino acid residues that could be part of a short linear binding motif. Some additional out-of-sequence 15mers were used to allow comparison between imperfectly-aligned AP180 and CALM sequences (See Figure A in S1 File). Specific amino acid residues were substituted with Ala to test the effect on binding. Peptide sequences, from the AP180 and CALM CLAP domains, with known CBMs were included as positive controls.

The peptide sequences were from the ADΔCLAP from mouse AP180 isoform 1 and 2 (identical; UniProtKB accession: Q05140; gene name: *Snap91*) and human CALM isoform 3 (UniProtKB accession: Q13492; gene name: *PICALM*). CALM isoform 3 was selected since it has the longest ADΔCLAP. CALM isoform 1 was used for all other experiments. The membrane was rinsed in methanol initially for 5 min to solubilise hydrophobic peptides. Then, the membrane was washed 3 x 10 min with Tris buffered saline spiked with Tween 20 (T-TBS: 137 mM NaCl, 2.7 mM KCl, 50 mM Tris-HCl pH 8.0, with 0.05% Tween 20), and blocked for 16 h at 4°C with agitation in 3% bovine serum albumin (BSA)/T-TBS. The membrane was incubated with purified clathrin from bovine brain (prepared as described previously [16]) at a concentration of 1.3 μg/ml in 3% BSA/T-TBS for 4 h. The membrane was washed 4 x 5 min with T-TBS, and incubated with primary antibody (1:1,000) in 3% BSA/T-TBS for 1 h. After repeating the wash procedure, secondary antibody was added at 1:20,000 in 3% BSA/T-TBS for 1 h. After washing in 3 x T-TBS, the membrane was incubated with chemiluminescence reagent and the luminescence was measured. The intensity of each spot was determined after subtraction of the background (a part of the membrane without a peptide spot).

### Production of plasmids and bacterial protein expression

Mouse AP180 isoform 2 was cloned into pGEX-6P-1, as described previously [12]. Human CALM isoform 1 (NCBI reference sequence NM_007166.3 for *PICALM*) was *de novo* synthesised and cloned into pGEX-6P-1 (using BamH1 and Not1) and pEGFP-C1 (using Sal1 and Xma1) by GenScript. AP180 and CALM mutants were generated by a commercial service (GenScript). Mouse AP180 isoform 2 Site 1 mutant consisted of L750A + L754A + L757A + L761A + I763A. Mouse AP180 isoform 2 Site 2 mutant consisted of L887A + L890A + I892A + F895A + L896A. Human CALM isoform 1 Site 1 mutant consisted of L540A + L544A + L547A + L551A + I553A. Human CALM isoform 1 Site 2 mutant consisted of I649A + F651A + M652A. Plasmid sequences were confirmed by a commercial sequencing service (Australian Genome Research Facility) after transformation. Transformations were performed in JM109 competent *Escherichia coli* cells (50 μl) with 25 ng DNA. A single colony containing transformed DNA was selected. Cultures were grown in Lysogeny Broth media containing 100 μg/ml ampicillin. Glutathione S-transferase (GST) fusion protein expression was induced with the addition of 1 mM IPTG and grown for a further 4 h, then pelleted at 10,000 rpm for 15 min at room temperature (~23°C) in a Beckman Coulter JLA-10.500 fixed angle rotor. Each protein pellet from 500 ml of culture was resuspended in 40 ml salt-Tris-EDTA buffer (300 mM NaCl, 10 mM Tris pH 8.0 and 1 mM EDTA), to which was added 1 complete EDTA-free protease inhibitor cocktail tablet. The solution was then made up to 1% Triton X-100, 1 mM DTT, 1 mM phenylmethylsulfonyl fluoride, 10 μg/ml leupeptin, 1 mM benzamidine and 5 μg/ml pepstatin A. Lysozyme was added to a final concentration of 0.1 mg/ml, and incubated on rotation at 4°C for 30 min. The pellets were subjected to two freeze-thaw cycles and probe sonication. The resulting lysate was centrifuged at 20,000 rpm for 20 min in a Beckman Coulter JA-25.50 fixed angle rotor. The supernatant was collected and stored on ice. Glutathione-Sepharose beads were prepared by washing in 0.1% Triton X-100/PBS three times, and incubated with the same solution for 30 min with rotation at 4°C. A 1 ml packed volume of beads was added to the collected lysate. The beads were incubated with the lysate for 1 h with rotation at 4°C. The beads were washed with 40 ml of ice cold buffers in the following order: 1) 1 wash with PBS; 2) 2 washes with 1.5 M NaCl in PBS; 3) 1 wash with 0.1% Triton X-100; 4) 3 washes with PBS. A final 2 ml of 50% slurry of approximately 1 mg/ml suspension of immobilised fusion protein was produced.

### Protein extraction

Beads with bound GST fusion protein were added to a spin-column (Illustra MicroSpin Columns, 50–700 μl capacity, GE Healthcare Life Sciences) and were washed with 4 x 500 μl ice cold PBS before addition of synaptosome lysate (11–13 mg/ml). Lysate was prepared from P2 synaptosomes prepared from rat brain as described previously [12]. All animal care and use complied with local legislation and procedures were approved and performed in accordance with institutional guidelines (Children’s Medical Research Institute/Children’s Hospital Westmead Animal Ethics Committee). Typically, 5 μg of bait protein was used in a pull-down with 2–2.5 mg of protein in the lysate. The columns were incubated for 10 min or 1 h in synaptosome lysate at 4°C with rotation. The lysate was removed by centrifugation and the beads were washed 3 x in a solution of 20 mM Tris pH 7.4, 1 mM EGTA, 1 mM EDTA with dissolved complete EDTA-free protease inhibitor cocktail tablet (1 tablet/20 ml). The bound proteins were eluted in SDS-PAGE sample buffer with heating for 5 min at 85°C. Proteins were resolved by SDS-PAGE in 1 mm thick 7.5 to 15% gradient large gels (20 cm) or mini-gels (Protean II or MiniProtean, Bio-Rad) before either Coomassie staining or transferring to nitrocellulose for Western blotting. Blots were recorded by exposure in an ImageQuant LAS 4000 digital chemiluminescence reader (GE Healthcare) for the appropriate exposure time and analysed in MultiGauge version 3.0.0.0 (Fujifilm Global). The final amount of bound protein was adjusted according to the amount of fusion protein bait detected by Coomassie staining. A one-way ANOVA followed by Tukey’s multiple comparison test was used to determine statistically significant differential binding.

### GST-tag removal

GST-tagged human rhinovirus 3C protease was expressed in *Escherichia coli*, as above. The beads were transferred to a 10 ml Bio-Rad Poly-Prep Chromatography Column. This column was washed with 50 mM Tris-HCl pH 8, 150 mM NaCl, 1 mM DTT supplemented with 0.01% Triton X-100 and then with the same solution containing 25 mM reduced glutathione at 4°C. The liquid was eluted and dialysed (membrane MW cut-off 12–14 kDa) against 1 L of TBS with 1 mM DTT for 20 h at 4°C with a buffer change at 4 h. The dialysed protease was collected, diluted with an equal volume of glycerol before being aliquoted and stored at -80°C.

GST-AP180 fusion proteins beads were washed in 150 mM NaCl, 1 mM EDTA, 1 mM DTT, 0.01% Triton X-100 and 50 mM Tris-HCl pH 7.0. The recombinant human rhinovirus 3C protease was added (10 μl protease/200 μl packed bead volume) and the spin-column was incubated and rotated at 4°C for 16 h. The cleaved protein was eluted, aliquoted and stored at -80°C. The eluted AP180 solution was dialysed against a 1 L solution of 0.5 M Tris pH 8 / 1 mM DTT for 20 h at 4°C with a buffer change at 4 h. Frozen pellets (drops from a glass Pasteur pipette into liquid nitrogen) were prepared immediately after dialysis and stored at -80°C.

### Clathrin Assembly Assays

Purified clathrin and AP180 protein stocks were dialysed at 4°C into 10 mM Tris pH 8.0 (with 2 mM DTT added to the clathrin solution). The concentration of each protein was determined by spectrophotometric measurement of absorbance at 280 nm. The purified clathrin stock was 3.75 μM and was used directly. The AP180 and CALM proteins were diluted to a working stock of 10 μM in 10 mM Tris pH 8.0. A light scattering assay [17] was performed with a DynaPro PROTEIN SOLUTIONS dynamic light scattering instrument (Wyatt). Data was acquired using the DYNAMICS 7.1.7 software. For all experiments it was confirmed that clathrin cages were formed in the 20 to 80 nm diameter range and that no larger aggregates were formed. The laser power was set at 30% and operated at 10 Hz for all experiments. Clathrin and AP180 concentration was fixed at 0.5 μM and 0.54 μM, respectively. Clathrin was added to a solution of AP180 and 10 mM Tris-HCl pH 8.0, which was equilibrated at room temperature for 1 min and then added to the cuvette which contained an aliquot of 1 M MES pH 6.7. The final concentrations of Tris and MES were 9 mM and 100 mM, respectively. The intensity of scattered light was averaged and recorded every 10 s. The data was processed using Origin Pro 9.1 (OriginLab). A baseline for assembly was established by adding the amount of light scattered by clathrin alone to the amount scattered by WT AP180. The intensity of scattered light for the assembly experiments was divided by this baseline level. A double exponential association curve was fitted to the assembly data, after constraining the baseline to equal 1, to obtain an initial rate of assembly parameter.

### Transferrin Uptake Assay

DMEM was supplemented with 10% FBS and used to maintain COS-7 cells at 37°C in a humidified atmosphere of 5% CO_2_. Confluent COS-7 cells were resuspended and diluted in DMEM/10% FBS and seeded in 12 well plates (containing glass coverslips coated with poly-D-lysine) at ~80,000 cells/well, and left in the incubator for 24 h. DNA (1 μg) and Fugene transfection reagent (3 μl) were mixed in 500 μl DMEM/10% FBS. The mixture was left to stand at room temperature for 20 min. The DNA-Fugene complexes were added drop wise to each well (500 μl/well) and the dishes gently swirled. The dishes were returned to the incubator for 3 h, and then topped up with 1 ml DMEM/10% FBS per well and returned to the incubator. Transfected cells were used in Tfn uptake assays 24 h post-transfection. Each plasmid was transfected in two wells for each independent experiment.

The cells were serum starved in DMEM without FBS for 4 h in the incubator. Tfn conjugated to Alexa Fluor 594 (5 μg/μl) was added and the cells were placed back in the incubator for 10 min. Tfn containing medium was immediately aspirated, washed 3 x with warm PBS (after 5 min incubations in PBS) and fixed with 4% paraformaldehyde for 15 min at room temperature, followed by 3 more PBS washes. Coverslips were mounted on slides spotted with DABCO to stain the nuclei and help the coverslip stick to the slide; the coverslip was sealed around the edges with clear nail polish to avoid drying out of the fixed cells, and the slides were stored in the dark at 4°C until imaging. The cells were visualised using an Olympus IX81 motorized inverted microscope (Hamamatsu Photonics) and images captured in three channels: red for Tfn (exposure 800 ms) and green for AP180 or CALM (exposure 200 ms). Metamorph software was used to calculate average intensity (AI) of Tfn uptake per cell, with the transfected cells normalised to non-transfected control cells (a minimum of 30 cells quantified per condition):

$$Tfn\;uptake\;AI=\frac{Transfected\;cell\;AI-Background\;AI}{Non-transfected\;cell\;AI-Background\;AI}$$

The average and SEM from three independent cell cultures were calculated using Prism 5 (GraphPad Software). Statistical analysis was done using a one-way ANOVA followed by Tukey’s multiple comparison test. GFP-tagged WT and mutant expression of AP180 and CALM was determined by lysis, SDS-PAGE and Western blotting with anti-GFP (Roche, 1:1000). Anti-β-actin (Sigma, 1:50,000) was used to demonstrate equal protein content in the lysates.

## Results

### Identification of sites of clathrin binding in the AP180 and CALM ADΔCLAP domain

**Fig 1:**
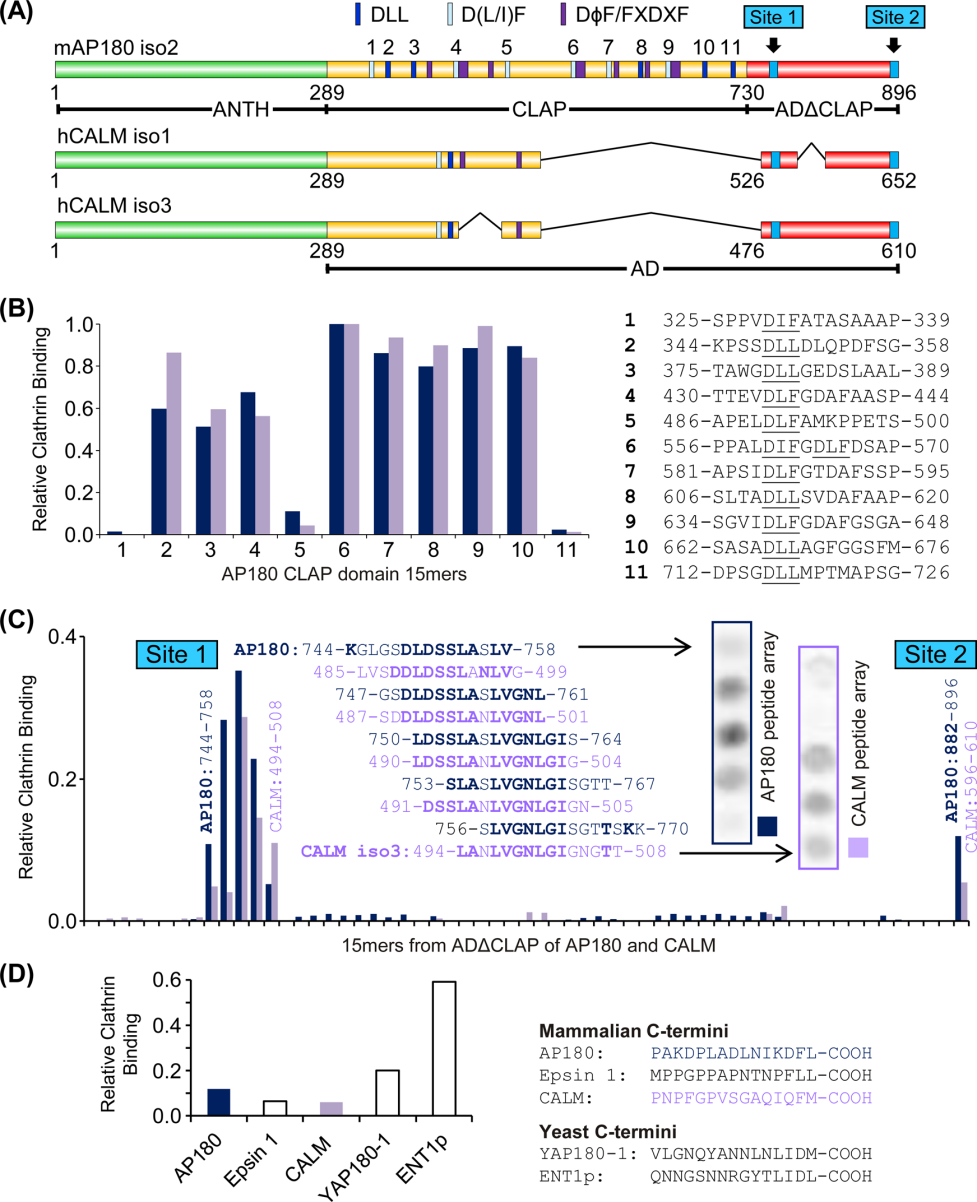
Binding of clathrin to 15mer peptides from the AP180 CLAP domain and AP180 and CALM ADΔCLAP domains. **(A)** Diagram indicating AP180 and CALM domains, the location of existing clathrin heavy chain (CHC) and adapter protein complex 2 (AP2) binding motifs [7] and the clathrin binding sites identified in this work. Note CALM isoform 3 was used to obtain results in this figure and Fig 2. CALM isoform 1 was used in subsequent figures. **(B)** The relative amount of purified clathrin bound to AP180 15mers containing known CBMs in an overlay assay. Numbers correspond to numbered binding motifs in (A). The dark blue columns and purple columns correspond to the overlay experiment with the AP180 and CALM ADΔCLAP 15mers, respectively. The intensity for each peptide was divided by the intensity of clathrin binding to peptide 6. **(C)** The amount of clathrin binding to overlapping 15mer peptides from the ADΔCLAP of AP180 isoform 2 and CALM isoform 3 (see Figure A in S1 File for full list of aligned peptides), relative to peptide 6 in (B). Same colour scheme as (B). Sequences and spots for Site 1 are shown. Bold residues in sequence alignment indicate identity between AP180 and CALM. The respective C-termini from AP180 and CALM are both referred to as Site 2. **(D)** Clathrin binding for AP180 and CALM Site 2 15mers compared to 15mer peptides from the C-termini of other clathrin adaptors (normalized to peptide 6 in (B), *n =* 1).

The specific sequences within the AP180 and CALM ADΔCLAP (Fig 1A) which bind clathrin were isolated using an overlay assay of an array of peptides, created using “spot synthesis”. Fifteen amino acid residue length peptides from the ADΔCLAP of AP180 and CALM were synthesised and immobilised on cellulose membrane. Each peptide overlapped with the adjacent peptide by 12 amino acid residues, and each peptide differed from the adjacent peptide by 3 amino acids. Additional peptides that differed by less than 3 amino acids were also included to improve the alignment of CALM with AP180. The CALM isoform 3 (CALM iso3) sequence was used for the array, since isoform 3 has the longest ADΔCLAP (Fig 1A). Purified clathrin was incubated with the membrane and bound clathrin was detected by Western blotting. The ADΔCLAP of AP180 and CALM were examined in two separate experiments. Each experiment had the same series of AP180 CLAP domain peptides, which served as positive controls and enabled the spot intensity for the two experiments to be compared (Fig 1B). Thus, the two experiments were designed to localise sequences in the ADΔCLAPs of AP180 and CALM that account for the ability of this sub-domain to bind clathrin. These peptide array assays are typically not repeated, i.e. *n* = 1 is typical [18,19]. However, the design ensured that the positive control peptides were examined twice. Furthermore, the results were validated by subsequent binding studies and functional experiments (see below).

The relative binding of clathrin to the AP180 CBMs has not been systematically examined. Therefore, these peptides are not only important positive controls, but provide novel insight on the relative binding strength of AP180 CBMs. The 11 peptides from the AP180 CLAP domain (Fig 1B) contained at least one CBM of type D(L/I)(L/F). Peptides 2, 3, 4, 6, 7, 8, 9 and 10 bound clathrin well above background in the two independent experiments. A sequence similar to peptide 3 was previously show to be able to block endocytosis *in vivo* [7]. Also, a sequence containing peptides 9 and 10 was shown in precise detail to use their DL(L/F) motifs to bind directly to the NTD [9]. Thus, these three peptides contain well-characterized NTD binding motifs. In contrast, the data shows that the D(L/I)(L/F) motifs in peptides 1, 5 and 11 had a relatively low level of clathrin binding (Fig 1B). Thus, the AP180 CLAP domain likely has functional and non-functional CBMs.

The overlay assay of the ADΔCLAP peptides revealed two corresponding sequences in AP180 and CALM that bound clathrin above the background level. The AP180 and CALM ADΔCLAP peptides were approximately aligned in Fig 1C so they could be readily compared (see Figure A in S1 File for complete list of peptide sequences). The first ADΔCLAP site that bound clathrin, hereafter “Site 1”, was moderately Leu/Ile-rich (Fig 1C). The peptide sequences with the highest affinity were LDSSLA[S/N]LVGNLGI[S/G] (square brackets show AP180, then CALM residue for that position). This sequence does not contain any canonical short linear motifs known to bind clathrin.

The second site that bound clathrin, hereafter “Site 2”, was located in the extreme C-terminus of both CALM and AP180 (Fig 1C and 1D). The level of clathrin binding was relatively weak. These sequences are neither conserved between CALM and AP180, nor do they contain CBMs. However, both extreme C-termini have adjacent hydrophobic amino acid residues (hCALM isoform 3, 609-FM-610 (isoform 1, 651-FM-652); mouse AP180 isoform 2, 895-FL-896) and the AP180 C-terminus has some nearby acidic amino acid residues, which are known features of CBMs [4,7].

The C-termini of yeast AP180 and epsin homologs, YAP180-1 and Ent1p, contain clathrin box motifs where the C-terminal carboxyl group suffices for the last polar group [20]. We compared the clathrin binding ability of mammalian and yeast homologs in an overlay assay. The C-termini of mammalian monomeric adapters AP180, epsin 1 and CALM bound qualitatively less clathrin than the yeast homologs (Fig 1D).

**Fig 2:**
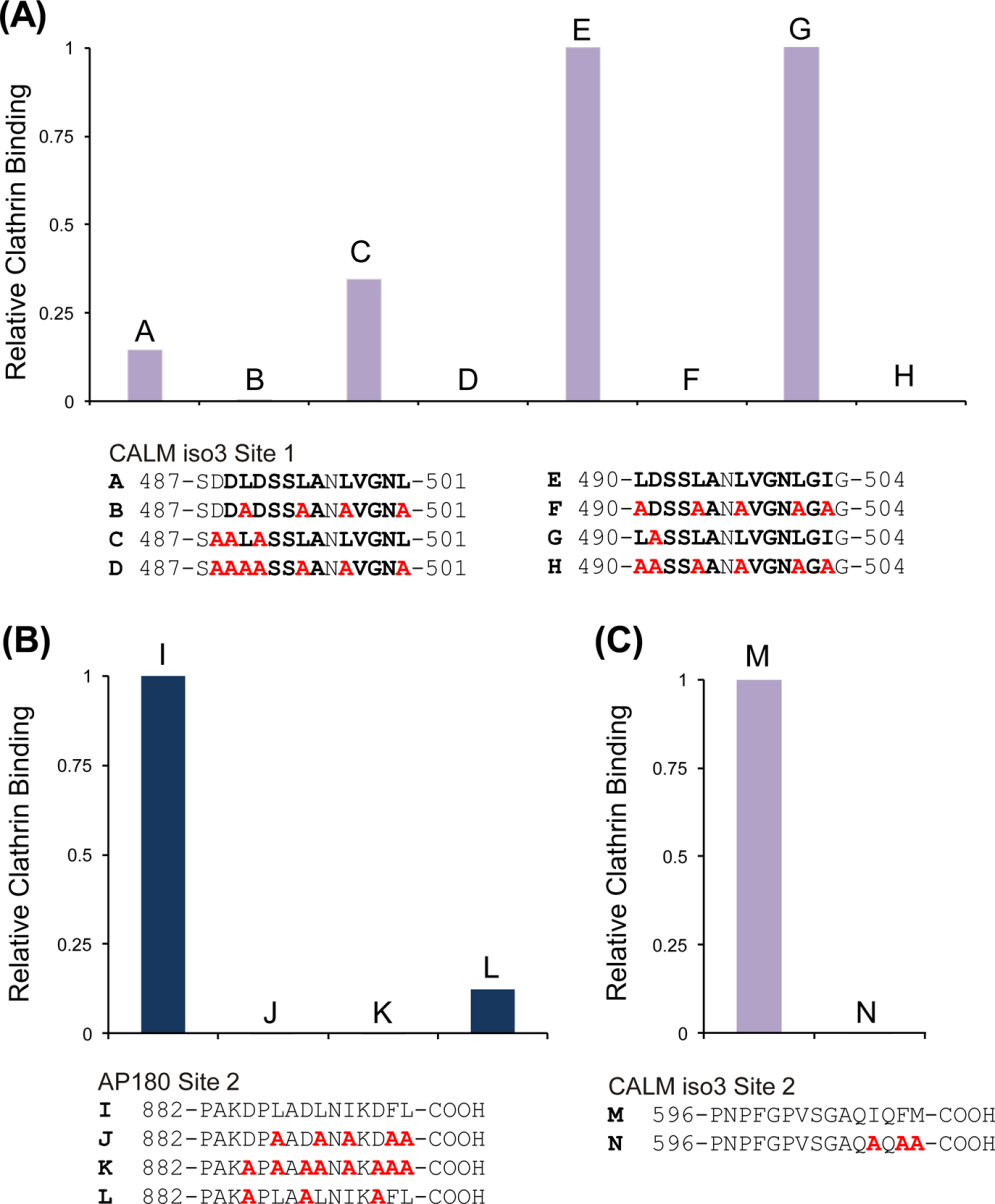
Substitution of amino acid residues in peptides from Site 1 and Site 2 to identify those important for clathrin binding. Hydrophobic amino acids (Leu, Ile, Met, and Phe), Asp residues and conserved residues in the extreme C-terminus were substituted with Ala (red residues) in peptide array overlay assays with purified clathrin. Bold residues indicate conserved amino acids. **(A)** CALM Site 1 substitutions and their effect on clathrin binding to 15mers (*n =* 1). The intensity for each 15mer has been divided by the intensity of clathrin binding to 15mer E **(B)** AP180 Site 2 substitutions and their effect on clathrin binding to 15mers (*n =* 1). The intensity for each 15mer has been divided by the intensity of clathrin binding to 15mer I **(C)** CALM Site 2 substitutions and their effect on clathrin binding to 15mers (*n =* 1). The intensity for each 15mer was divided by the intensity of clathrin binding to 15mer M.

To determine the amino acid residues in Site 1 and Site 2 important for clathrin binding, we used peptides with alanine substitutions at specific residues in the sequences. Conserved, acidic and hydrophobic residues were targeted, since these residues occur in known CBMs and these sequences are likely to have a conserved function. Substitution of all the conserved Leu/Ile residues with Ala in CALM iso3 490-LDSSLANLVGNLGIG-504 prevented almost all clathrin binding (Fig 2A, *c*.*f*. peptides 2E and 2F). Removal of Asp residues alone had no effect (Fig 2A, *c*.*f*. peptides 2E and 2G) and no additional effect could be observed by removing all Leu/Ile in combination with all Asp residues since the Leu/Ile mutation caused bound clathrin to be already at background level (Fig 2A, *c*.*f*. peptides 2E and 2H). The same effect was observed for CALM iso3 487-SDDLDSSLANLVGNL-501 (Fig 2A peptides 2A to 2D), which bound less clathrin than CALM iso3 490–504, but independently verified the importance of Leu/Ile to Ala mutation. We did not separately test if these mutations had the same effect on clathrin binding to an AP180 Site 1 15mer, since these same Leu/Ile residues were 100% conserved.

Hydrophobic residues (3 Leu, 1 Ile and 1 Phe) were substituted for Ala in AP180 Site 2 peptide 882-PAKDPLADLNIKDFL-896 and this was sufficient to block clathrin binding (Fig 2B). When the three Asp residues were substituted with Ala, binding of clathrin decreased, similar to the substitution of hydrophobic residues, indicating that acidic residues may have a role in clathrin binding. For the CALM iso3 C-terminus peptide, 596-PNPFGPVSGAQIQFM-610, three hydrophobic residues within the last four residues were substituted (Ile, Phe and Met) for Ala and clathrin binding was abolished (Fig 2C). Thus, clathrin binding to AP180 Site 1 is dependent on Leu/Ile residues, CALM Site 2 is dependent on hydrophobic residues and AP180 Site 2 is dependent on both hydrophobic and acidic residues. This latter observation supports the hypothesis that the extreme C-terminus of AP180 might be a degenerate CBM that binds the clathrin NTD.

### The effect of Site 1 and 2 mutation on the extraction of clathrin from brain lysate

**Fig 3:**
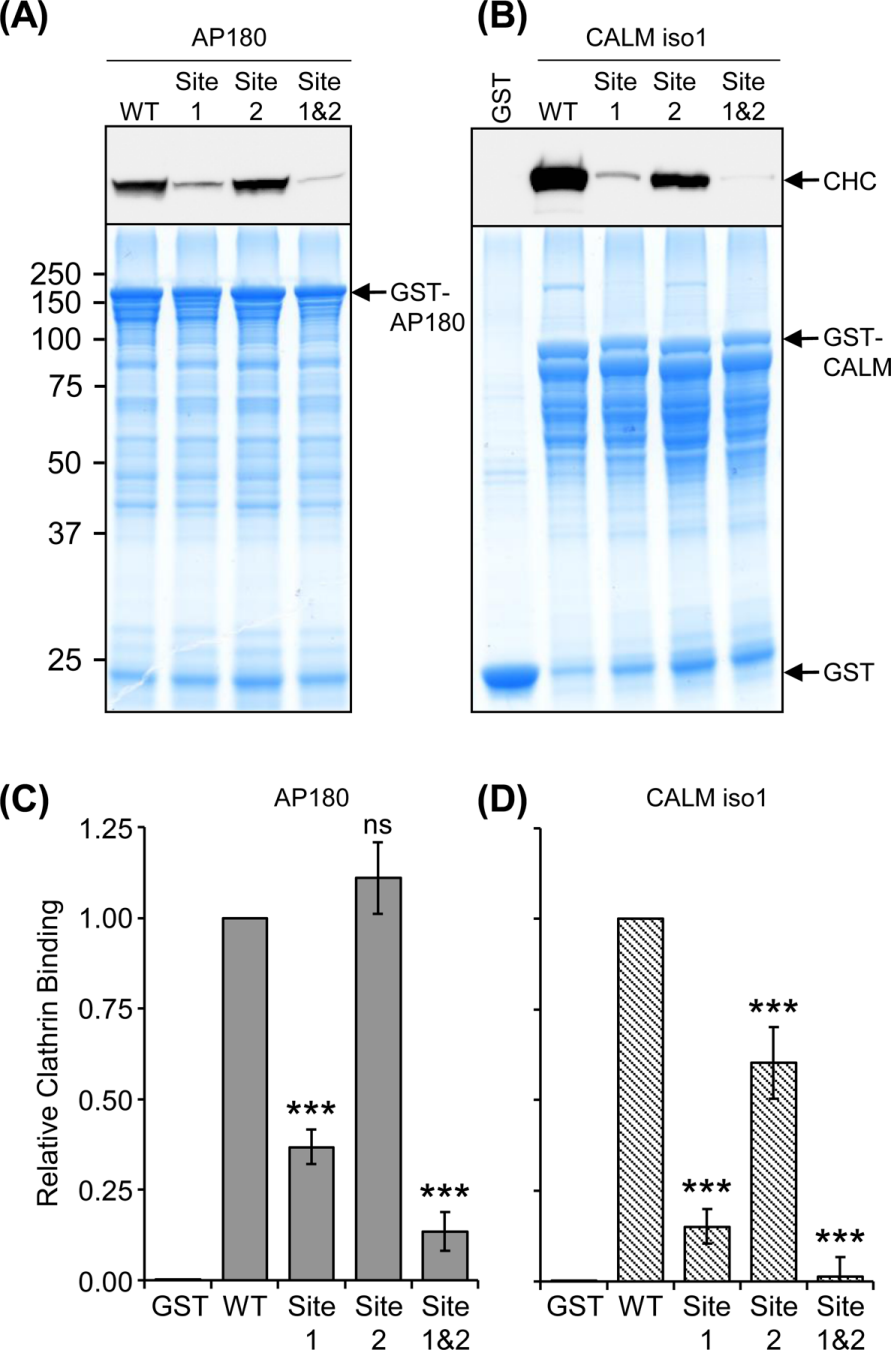
Extraction of clathrin from synaptosome lysate by GST-tagged WT, Site 1 and 2 mutated AP180 and CALM. **(A)** AP180 and **(B)** CALM mutated at Site 1, Site 2 and Site 1&2 (double mutant) were used in 1 h pull-downs with rat synaptosome lysate and the amount of clathrin bound was determined by Western blot with anti-clathrin heavy chain (CHC) using 40% of the sample (representative blot shown from two 1 h pull-down experiments). Ten percent of the sample was resolved by SDS-PAGE and stained with Coomassie for comparison of bait levels. The experiment was performed twice more, except the pull-down was for 10 min instead of 1 h and the result was very similar (Figure B in S1 File). **(C)** and **(D)** Comparison of clathrin binding from the combined densitometry of 10 min and 1 h pull-downs with the AP180 mutants normalised to AP180 WT and the CALM mutants to CALM WT. Data is expressed as average relative amount of clathrin bound as a fraction of the WT pull-down ± SEM (*n =* 4; ***, *P* < 0.001 compared to WT; ns = not significant).

The conserved Leu/Ile residues in AP180 and CALM Site 1 and the crucial hydrophobic residues identified above for each Site 2 sequence were mutated (See Materials and Methods and Figure A in S1 File for specific residues) to determine whether these sequences affect the efficiency of clathrin binding in a pull-down experiment (Fig 3). Note that these mutations and subsequent experiments were performed using the CALM isoform 1 sequence, not isoform 3 (Fig 1A). The pull-down with GST-tagged sequences was done using both 1 h (Fig 3A and 3B) and 10 min durations (Figure B in S1 File) to potentially show kinetic information. However, the results for these time points were indistinguishable and so they were combined in a quantitative analysis of clathrin binding (Fig 3C and 3D). Mutation of Site 1 greatly reduced clathrin binding in both AP180 and CALM compared to the WT. Mutation of Site 2 only reduced clathrin binding in CALM (Fig 3B and 3D, *P* < 0.001 for all three mutants, comparing to the wild type, *n =* 4). The combined mutation, Site 1&2, further reduced clathrin binding for CALM (Fig 3B and 3D, CALM Site 1 vs Site 1&2, *P* < 0.001). The AP180 Site 1&2 mutant was not significantly reduced compared to S1. However, both AP180 and CALM S1&2 mutants did not bind clathrin significantly different to the non-specific binding of GST alone, whereas Site 1 alone was significantly different to GST alone for both AP180 (*P* < 0.01) and CALM (*P* < 0.001). The more pronounced effect of Site mutation for CALM could be due to an overall lack of CBMs compared to AP180. We conclude that Site 1 was the major ADΔCLAP binding site and that, at least for CALM, both Site 1 and 2 contributed to efficient clathrin extraction.

### The effect of AP180 Site 1 and Site 2 mutation on clathrin assembly

**Fig 4:**
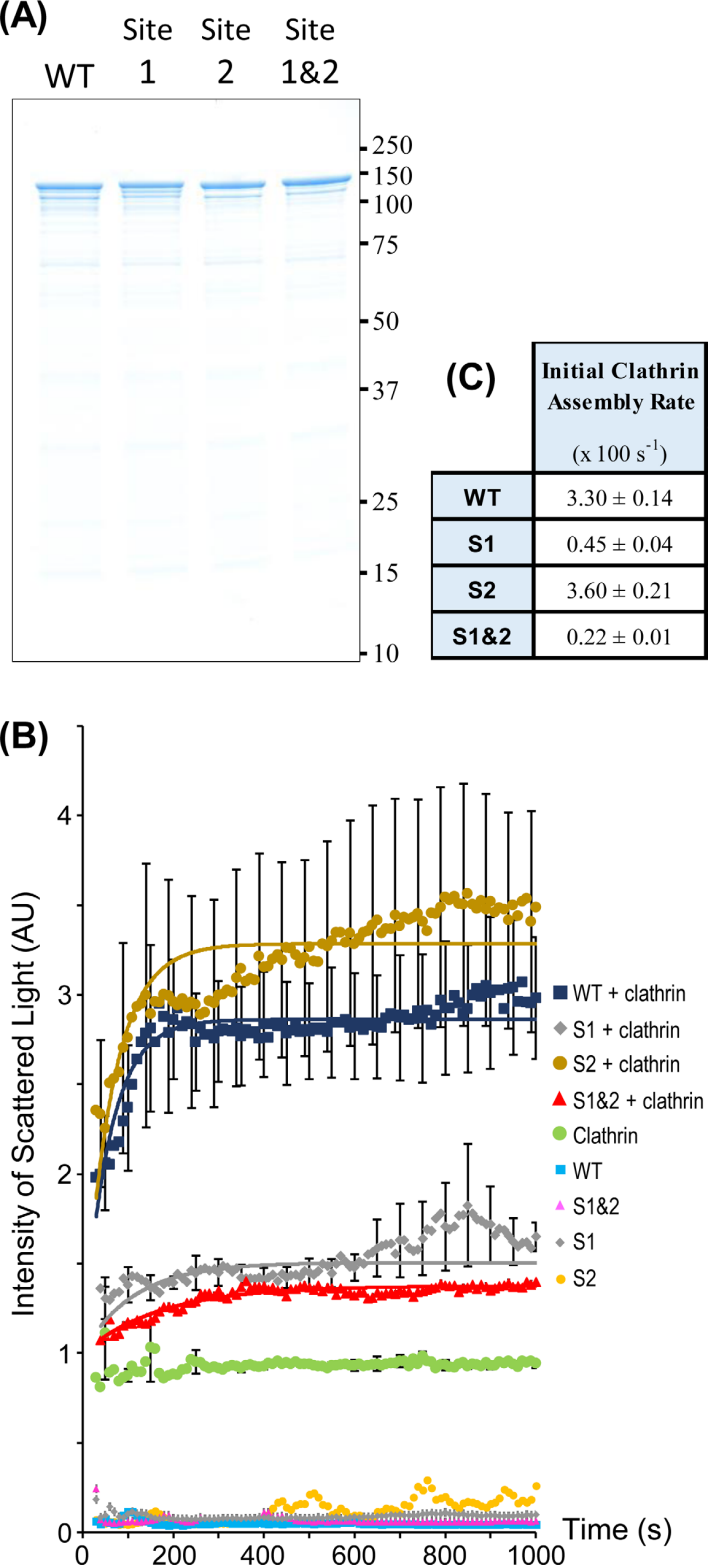
Comparison of clathrin assembly by WT, Site 1 and Site 2 mutated AP180. **(A)** A Coomassie stained SDS-PAGE gel of WT AP180, Site 1 (S1), Site 2 (S2) and Site 1&2 (S1&2) mutants, which were bacterially expressed and the GST-tag removed. **(B)** Light scattering was used to determine the clathrin assembly rate for fixed concentrations of purified AP180 and clathrin. Data shown is the mean ± SEM (*n =* 5 for experiment which used clathrin, *n* = 2 for the remaining controls). Only every 5^th^ error bar is shown. The S2 + clathrin error bars are staggered by 10 s to improve visual clarity. Data was normalised to baseline assembly (the sum of scattered light intensity from clathrin alone plus WT AP180 alone = 1). Curves were fitted to calculate initial rates of clathrin assembly (lines). **(C)** Initial clathrin assembly rates determined from curve fitting of assembly data in (B). Errors for these parameters are SEM (*n =* 5).

An *in vitro* clathrin assembly assay was used to measure the effect of AP180 ADΔCLAP mutation on clathrin cage assembly. GST-tags were cleaved from the bacterially expressed AP180 sequences and the untagged proteins were purified (Fig 4A). Purified bovine brain clathrin was mixed with WT or mutant AP180 and then cage assembly was triggered by a decrease in pH (see Materials and Methods) [21]. An assembly assay using light scattering allowed an assessment of the kinetics of assembly at a fixed concentration of AP180 (0.5 μM). AP180 Site 1, Site 2, and Site 1&2 mutants were compared to AP180 WT (Fig 4B). Curves were fitted to the data to enable calculation of the initial rate of assembly (Fig 4C). The Site 1 mutant had a 7 to 8-fold slower initial rate of assembly compared to AP180 WT. The AP180 double mutant (Site 1&2) was 15-fold slower than WT. The Site 2 initial rate of clathrin assembly was similar to WT (Fig 4C). The same assembly experiment was performed using WT and mutated CALM, however, the extent of clathrin assembly was very low, preventing sensitive comparison of assembly rates (data not shown). In summary, the AP180 Site 1 mutant demonstrated a deficit in the initial rate of assembly of clathrin cages.

### The effect of Site 1 and Site 2 mutants on transferrin uptake

**Fig 5:**
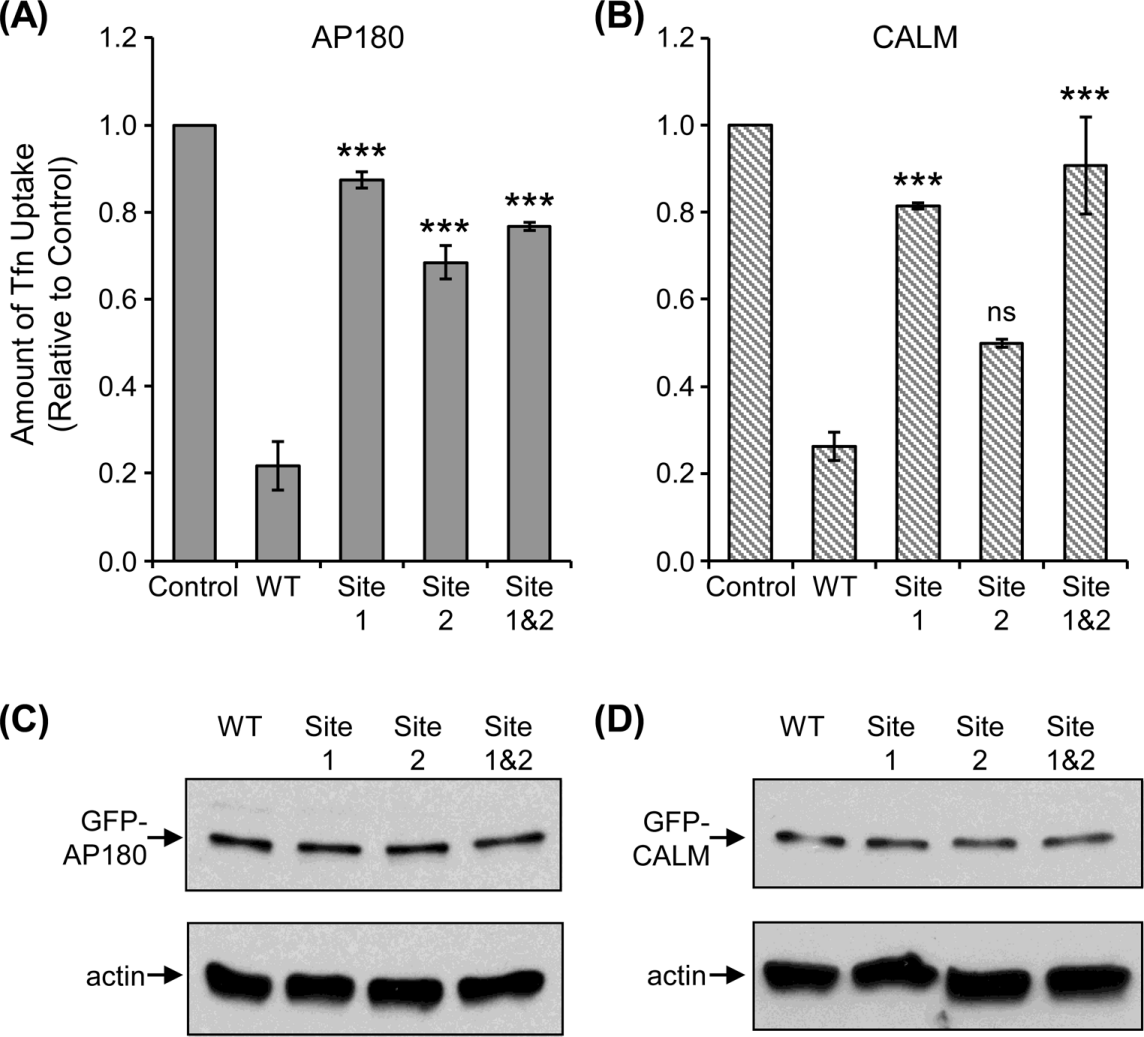
Comparison of WT, Site 1 and Site 2 mutated AP180 and CALM in a transferrin uptake assay. COS-7 cells were transfected with GFP-tagged WT, Site 1, Site 2 and Site 1&2 mutants for **(A)** AP180 and **(B**) CALM. Alexa Fluor 594 labelled Tfn (red) was added and the amount of uptake was measured as an assay of endocytosis function and clathrin sequestration. Note that in (A) and (B), respectively, GFP-tagged WT AP180 and CALM exhibit a dominant negative effect due to sequestration of clathrin. Data is expressed as a fraction of Tfn uptake in untransfected control cells ± SEM (*n =* 4; ***, *P* < 0.001 compared to WT; ns = not significant). The approximately equal expression level of AP180 and CALM fusions with GFP is shown in panels **(C)** and **(D)**, respectively, which was produced from COS-7 cell lysis, SDS-PAGE and Western blotting with anti-GFP or anti-actin (control for protein content).

Since both AP180 and CALM participate in endocytosis, a ligand-receptor uptake assay was done using Tfn conjugated to Alexa Fluor 694, which was incubated with variously transfected cells for 10 min. AP180 is not involved in receptor-mediated endocytosis, however, CALM is specifically involved in Tfn receptor endocytosis [15,22]. Tfn uptake in GFP-WT AP180 or CALM transfected cells was compared to uptake in the presence of GFP fusions of Site 1, Site 2 and the Site 1&2 double mutant AP180 (Fig 5A and 5B, see Figures C and D in S1 File for representative microscope images). All proteins were confirmed to be similarly expressed in COS-7 cells and not subject to rapid degradation (Fig 5C and 5D). WT AP180 and CALM had a dominant negative effect when transfected into COS-7 cells due to clathrin sequestration, as established in previous publications [12,14,15,23,24]. Control cells were not transfected. For both AP180 and CALM, the Site 1 mutants and Site 1&2 double mutants had similar uptake to the control cells (Fig 5A and 5B), indicating that mutation of these sites inhibited clathrin sequestration. Site 2 also reduced sequestration of clathrin in AP180, relative to WT, but not when the corresponding sequences were mutated in CALM. This may reflect the relative strength of clathrin binding for Site 2 in each of these proteins (see Fig 1C and 1D). Therefore, Site 1, in particular, in the ADΔCLAP of CALM and AP180 can have a significant role in clathrin binding in a cellular environment as demonstrated by the *ex vivo* Tfn uptake assay.

### A comparison of the effect of mutating clathrin binding motifs

**Fig 6:**
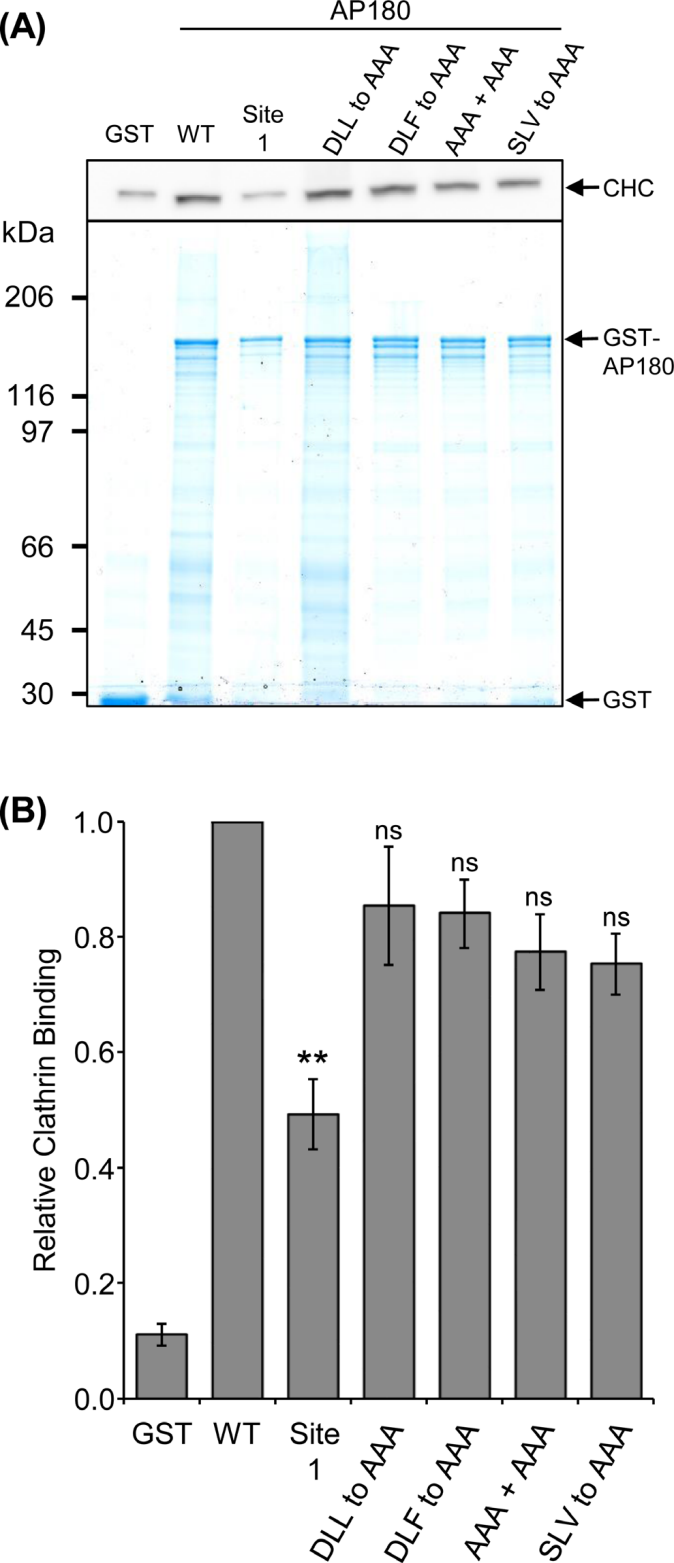
Extraction of clathrin from synaptosome lysate by GST-tagged AP180 WT, Site 1 and CBM mutants. **(A)** AP180 WT and the following mutant sequences, Site 1, 666-DLL-668 to AAA, 638-DLF-640 to AAA, both 666-DLL-668 and 638-DLF-640 to AAA (AAA+AAA) and 756-SLV-758 to AAA, were used in 1 h pull-downs with rat synaptosome lysate and the amount of clathrin bound was determined by Western blot with anti-clathrin heavy chain (CHC) using 25% of the sample. Five percent of the sample was resolved by SDS-PAGE and stained with Coomassie for comparison of bait levels. Representative Western blot and SDS-PAGE gel is shown from three 1 h pull-down experiments. **(B)** Comparison of clathrin binding from the densitometry of each 1 h pull-down with the AP180 mutants normalised to AP180 WT. Data is expressed as average relative amount of clathrin bound as a fraction of the WT pull-down ± SEM (*n =* 3; **, *P* < 0.01 compared to WT; ns = not significant).

The above experiments demonstrated that Site 1, in particular, has a large effect on the interaction of clathrin with AP180 or CALM. We investigated whether D(L/I)(L/F) site mutation would have a similar magnitude effect on clathrin binding, which has not been tested in full length AP180, until now. We mutated CBM sites in AP180 and compared the ability of GST-AP180 WT and mutants to extract clathrin from synaptosome lysate (Fig 6), similar to the experiment in Fig 3. Mutation of a single DLL motif (CBM 10, 666-DLL-668, Fig 1A and 1B) to AAA resulted in the extraction of an amount of clathrin that was not significantly different to WT AP180 (*n* = 3). Likewise, mutation of a DLF motif (CBM 9, 638-DLF-640, Fig 1A and 1B) to AAA did not significantly affect clathrin binding. The mutation of both 666-DLL-668 and 638-DLF-640 to AAA also did not affect binding (Fig 6). Therefore, mutation of single or double CLAP CBMs had no significant effect on clathrin binding.

We mutated five Leu/Ile residues within Site 1 without regard for a potential degenerate three-residue D(L/I)(L/F) CBM within this sequence (750-LDSSLASLVGNLGI-763). The most likely candidate for a three-residue CBM is the 756-SLV-758 sequence, since it has adjacent hydrophobic residues and Ser is occasionally found instead of Asp at the first position in longer CBMs [4,7]. Furthermore, this short sequence was present in every peptide that bound clathrin (above background) in Fig 1C. 756-SLV-758 was mutated to AAA and the binding was compared to WT AP180 (Fig 6). The amount of clathrin bound to the SLV to AAA mutant was not significantly different to WT indicating that this short sequence does not account for the effect of site 1.

## Discussion

Since CME relies on either AP180 or CALM for the formation of a clathrin coat, these two proteins are essential for regulated vesicle biogenesis. Here, we have partially determined why AP180 and CALM have sequence similarity in the C-terminal ~16 kDa and how this sequence affects clathrin binding and assembly. The ability of AP180 to assemble clathrin *in vitro* and the existence of a multitude of CBMs suggested that the AP180 CLAP domain has the required functional elements to form a clathrin coat on nascent synaptic vesicles. However, these D(L/I)(L/F) CBMs were not systematically tested for clathrin binding function, prior to this work. A peptide array overlay assay allowed the first examination of the relative binding of clathrin to each AP180 CLAP domain CBM. It was found that not all CBMs on AP180 are equal. Three of eleven CBMs bound relatively low levels of clathrin indicating they might not be functional. Since two previously studied AP180 CBMs did not change their localised structure whether free in solution or bound to the clathrin NTD [9], our results using cellulose-bound peptides are likely to be valid for the free protein and might indicate those CBMs used by clathrin *in situ*. If an arbitrary binding affinity threshold is applied, it is likely AP180 has only eight functional CBMs in the CLAP domain. How many CBMs does AP180 require? Contact between AP180 and multiple clathrin triskelia is required for assembly. AP180 has excess CBMs for this requirement and there is the assumption that abundant CBMs allow the formation of relatively small synaptic vesicles. The CALM CLAP has not been as thoroughly surveyed for non-canonical CBMs. The exact function of abundant CBMs within adapter proteins may become known as further mechanistic requirements for assembly, and the stages of coat maturation, are discovered.

The overlay assay identified two novel sequences in the ADΔCLAP of AP180 and CALM that bound clathrin. The results of the overlay assay were confirmed via binding, assembly and Tfn uptake assays. The first sequence, Site 1, is the major site. Site 2 is a minor site that was not confirmed as functional in all experiment types. Site 1 is conserved in AP180 and CALM and bound approximately half as much as the weaker CBMs from the CLAP domain. Site 2, which was not conserved but positioned at the extreme C-termini of each adapter, had much weaker binding and would not be considered a functional binding site if the same threshold, as above for a CBM, was applied. Clathrin binding to Site 1 was dependent on Leu/Ile only. The experiment using 756-SLV-758 to AAA mutation in AP180 confirmed that Site 1 was not harbouring a degenerate DL(L/F) type CBM. In contrast, Site 2 in AP180 was dependent on both hydrophobic and acidic residues, indicating (non-positional) similarity to classical CBMs. Site 2 in CALM was dependent on hydrophobic residues and had no acidic residues. These data demonstrate that Site 1 is a novel clathrin binding sequence, which allows the possibility that it binds to a non-NTD site on clathrin.

Site 1 mutation was sufficient to ablate the clathrin assembly function of AP180 and abolish the dominant negative Tfn uptake phenotype of overexpressed WT AP180 or CALM. This is the first time that mutation of a clathrin binding site has affected *in vitro* clathrin assembly. It is counterintuitive that a relatively weak clathrin binding sequence could have such a strong effect. The lack of measurable effect of mutated AP180 DL(L/F) sites in a pull-down experiment exemplified that Site 1 has a surprisingly large influence on clathrin binding, which was not predicted by the relatively low affinity of clathrin for isolated Site 1 peptides. These data suggest the proper function of the CLAP domain CBMs, which are undoubtedly essential for assembly, also requires the ADΔCLAP Site 1. Recent modelling of the role of adapter proteins in clathrin assembly [25] may provide an explanation for the role of Site 1. It was shown in simulations of assembly that the adapter affinity for clathrin triskelia needs to be sufficient to nucleate multiple triskelia, but assembly is inhibited by overly strong affinity. This is because of the inability of stronger binders to allow unbinding and reshuffling into progressively more cage-like configurations. Furthermore, the distance between two clathrin binding motifs/sequences is required to be within a certain range to favour inter-triskelion binding over intra-triskelion binding, which arose from modelling of binding to sites other than the NTD. The relatively low clathrin affinity of Site 1, the potential for Site 1 binding to a non-NTD site and the separation of Site 1 from highly clustered CBMs within AP180 (e.g., CBMs 6 to 10 are no more than 28 amino acid residues from the nearest CBM neighbour) may be advantageous for clathrin assembly. These hypotheses remain to be tested.

It is important to note that Site 1 overlaps with a nuclear export signal (NES) in CALM [26,27]. The NES consensus sequence is ΦX_1-3_ΦX_2-3_ΦXΦ, where Φ is most often Leu, is conserved within AP180 and CALM [3]. The NES is the CALM factor that contributes to CALM-AF10 mediated leukemogenesis [26–28]. An NES with a dual function was shown for 14-3-3 protein [29]. The sequence matching an NES in AP180 has not been shown to be functional and its mutation in this work did not result in nuclear export block (*c*.*f*. Figures C and D in S1 File). It is notable that the sequence adjacent to the AP180 750–763 appears to have an increased ability to bind clathrin, relative to the equivalent sequence in CALM (Fig 1C, *c*.*f*. AP180 747–761 to CALM iso3 487–501 and AP180 753–767 to either CALM iso3 491–505 or 494–508, Fig 2A, *c*.*f*. peptide 2A to 2C). This observation supports the hypothesis that the CALM NES has been adapted in AP180 to allow increased clathrin affinity by AP180 in SVE.

Site 2 in AP180 and CALM both have a di-hydrophobic residue motif at their C-termini. Extreme C-termini CBMs exist in yeast AP180 and epsin (YAP180-1 and Ent1p). The clathrin binding ability of these yeast extreme C-termini was compared to the termini of mammalian AP180, CALM and epsin 1. All mammalian C-termini were qualitatively weaker than the yeast epsin C-terminus. This data suggests an evolutionary gulf between the function of yeast and mammalian C-termini that may not have been entirely ablated. Site 2 in both AP180 and CALM did not have a consistent effect on clathrin binding in the experiment types presented here and so is less likely to be a functional binding site.

In conclusion, we have identified a novel clathrin binding site in the ADΔCLAP of AP180 and CALM. Thus, CALM now has at least two identified clathrin binding sites (including the lone DLL motif). The evidence here, and the original observation that the isolated ADΔCLAP sub-domain binds clathrin cages, suggests that Site 1 increases clathrin-AP180/CALM avidity, or provides an ideal pathway to regulated clathrin cage nucleation, and is essential for efficient cage assembly. This study has updated the role of the previously identified CBMs in AP180 and provided a sequence-specific explanation of clathrin-ADΔCLAP interaction.

## Supporting Information

S1 File Supporting Information (PDF)

## Abbreviations

AD: assembly domain
ADΔCLAP: the remainder of the AD after subtraction of the CLAP domain
AI: average intensity
AP180: assembly protein 180 kDa
BSA: bovine serum albumin
CALM: clathrin assembly lymphoid myeloid leukemia protein
CBM: clathrin binding motif
CLAP: clathrin and adaptor protein binding
CME: clathrin-mediated endocytosis
DMEM: Dulbecco’s Modified Eagle’s Medium
FBS: fetal bovine serum
hCALM: human CALM
GST: glutathione S-transferase
iso: isoform
mAP180: mouse AP180
MES: 2-(N-morpholino)ethanesulfonic acid
NES: nuclear export signal
NTD: clathrin heavy chain N-terminal domain
PBS: phosphate buffered saline
SEM: standard error of the mean
SVE: synaptic vesicle endocytosis
TBS: Tris buffered saline
T-TBS: Tris buffered saline with Tween 20
Tfn: transferrin
