# Supplementary material for: A Novel Sequence in AP180 and CALM Promotes Efficient Clathrin Binding and Assembly
Source: PLoS One. 2016 Aug 30;11(8):e0162050. doi: 10.1371/journal.pone.0162050 (PMC5004861; doi:10.1371/journal.pone.0162050)

Figure A. Peptide 15mers from the ADΔCLAP of AP180 and CALM used in the peptide array overlay assay. Peptides in Fig. 1 were plotted according in the order shown below. Peptides are from AP180 mouse isoform 2 and human CALM isoform 3. The alignment of sequences approximates previously published alignments (Morgan *et al.*, *J. Neurosci.*, 19, 10201-10212, 1999 and Moshkanbaryans, L. *et al.*, *Membranes*, 4, 2014). The 15mer for the CALM C-terminus was arbitrarily moved to the last position. Red residues indicate Site 1 and Site 2 amino acids that were mutated in subsequent experiments.

Order	AP180 ADΔCLAP 15mers	CALM ADΔCLAP 15mers	
1	720-PTMAPSGQPAPVSMV	467-TVASQNQNLPVAKLP	
2	723-APSGQPAPVSMVPPS	470-SQNQNLPVAKLPPSK	
3	726-GQPAPVSMVPPSPAM	473-QNLPVAKLPPSKLVS	
4	729-APVSMVPPSPAMAAS	476-PVAKLPPSKLVSDDL	
5	732-SMVPPSPAMAASKGL		
6	735-PPSPAMAASKGLGSD		
7	738-PAMAASKGLGSDLDS	479-KLPPSKLVSDDLDSS	
8	741-AASKGLGSDLDSSLA	482-PSKLVSDDLDSSLAN	
9	744-KGLGSDLDSSLASLV	485-LVSDDLDSSLANLVG	
10	747-GSDLDSSLASLVGNL	487-SDDLDSSLANLVGNL	
11	750-LDSSLASLVGNLGIS	490-LDSSLANLVGNLGIG	Site 1
12	753-SLASLVGNLGISGTT	491-DSSLANLVGNLGIGN	
13	756-SLVGNLGISGTTSKK	494-LANLVGNLGIGNGTT	
14		497-LVGNLGIGNGTTKND	
15	759-GNLGISGTTSKKGDL	500-NLGIGNGTTKNDVNW	
16	762-GISGTTSKKGDLQWN	503-IGNGTTKNDVNWSQP	
17	765-GTTSKKGDLQWNAGE	506-GTTKNDVNWSQPGEK	
18	768-SKKGDLQWNAGEKKL	509-KNDVNWSQPGEKKLT	
19	771-GDLQWNAGEKKLTGG	512-VNWSQPGEKKLTGGS	
20	774-QWNAGEKKLTGGANW	515-SQPGEKKLTGGSNWQ	
21	777-AGEKKLTGGANWQPK	518-GEKKLTGGSNWQPKV	
22	780-KKLTGGANWQPKVTP	521-KLTGGSNWQPKVAPT	
23	783-TGGANWQPKVTPATW	524-GGSNWQPKVAPTTAW	
24	786-ANWQPKVTPATWSAG	527-NWQPKVAPTTAWNAA	
25		530-PKVAPTTAWNAATMN	
26		533-APTTAWNAATMNGMH	
27		536-TAWNAATMNGMHFPQ	
28	789-QPKVTPATWSAGVPP	539-NAATMNGMHFPQYAP	
29	792-VTPATWSAGVPPQGT	542-TMNGMHFPQYAPPVM	
30	795-ATWSAGVPPQGTVPP	545-GMHFPQYAPPVMAYP	
31	798-SAGVPPQGTVPPTSS	548-FPQYAPPVMAYPATT	
32	801-VPPQGTVPPTSSVPP	551-YAPPVMAYPATTPTG	
33	804-QGTVPPTSSVPPGAG	554-PVMAYPATTPTGMIG	
34	807-VPPTSSVPPGAGAPS	557-AYPATTPTGMIGYGI	
35	810-TSSVPPGAGAPSVGQ	560-ATTPTGMIGYGIPPQ	
36	813-VPPGAGAPSVGQPGA	563-PTGMIGYGIPPQMGS	
37	816-GAGAPSVGQPGAGFG		
38	819-APSVGQPGAGFGMPP		
39	822-VGQPGAGFGMPPSGT		
40	825-PGAGFGMPPSGTGMT	566-MIGYGIPPQMGSVPV	
41	828-GFGMPPSGTGMTMMS	569-YGIPPQMGSVPVMTQ	
42	831-MPPSGTGMTMMSQQP	572-PPQMGSVPVMTQPTL	
43	834-SGTGMTMMSQQPVMF	575-MGSVPVMTQPTLIYS	
44	837-GMTMMSQQPVMFAQP	578-VPVMTQPTLIYSQPV	
45	840-MMSQQPVMFAQPMMR	581-MTQPTLIYSQPVMRP	
46	843-QQPVMFAQPMMRPPF	584-PTLIYSQPVMRPPNP	
47	846-VMFAQPMMRPPFGAA	587-IYSQPVMRPPNPFGP	
48	849-AQPMMRPPFGAAAVP	590-QPVMRPPNPFGPVSG	
49	852-MMRPPFGAAAVPGTQ	593-MRPPNPFGPVSGAQI	
50	855-PPFGAAAVPGTQLSP		
51	858-GAAAVPGTQLSPSPT		
52	861-AVPGTQLSPSPTPAT		
53	864-GTQLSPSPTPATQSP		
54	867-LSPSPTPATQSPKKP		
55	870-SPTPATQSPKKPPAK		
56	873-PATQSPKKPPAKDPL		
57	876-QSPKKPPAKDPLADL		
58	879-KKPPAKDPLADLNIK		
59	882-PAKDPLADLNIKDFL	596-PNPFGPVSGAQIQFM	Site 2

Figure B. Extraction of clathrin from synaptosome lysate by GST-tagged WT, Site 1 and 2 mutated AP180 and CALM, using a 10 min incubation. WT AP180 and CALM was compared to Site 1, Site 2 and Site 1&2 mutants in 10 min pull-downs with rat synaptosomes lysate (one representative blot and gel are shown from two independent experiments). Forty percent of the sample was loaded for SDS-PAGE followed by Western blot anti-clathrin heavy chain (CHC) and 10 % of the sample was loaded for SDS-PAGE followed by Coomassie staining (for comparison of bait levels). The result was very similar to the 1 h pull-down in Fig. 3.

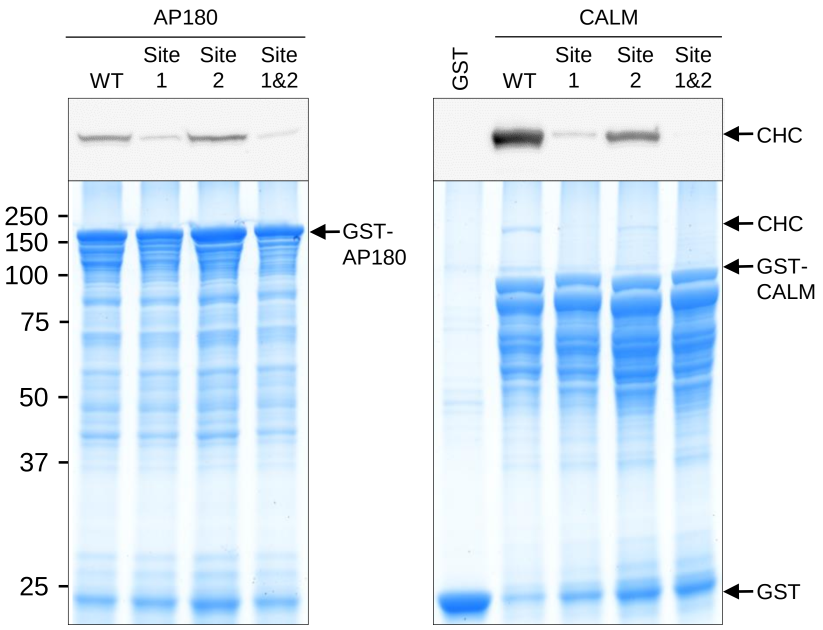

Figure C. Representative examples of GFP-AP180 WT and mutant transfected and control cells used in the transferrin uptake assay. GFP-AP180 WT and mutant proteins (green) were expressed in COS-7 cells and transferrin (red) was applied for 10 min. See Materials and Methods for experimental details.

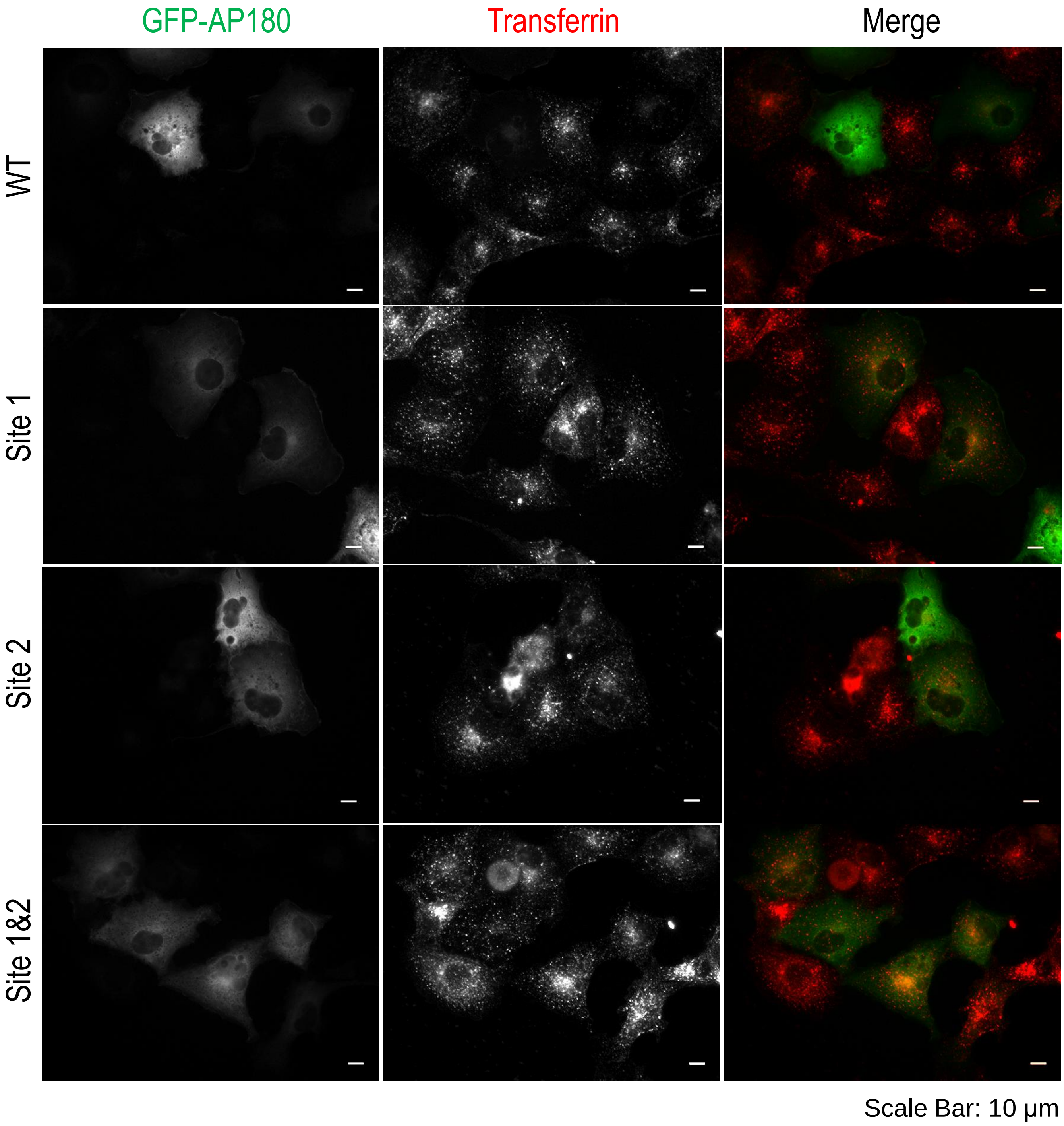

Figure D. Representative examples of GFP-CALM WT and mutant transfected and control cells used in the transferrin uptake assay. GFP-CALM WT and mutant proteins (green) were expressed in COS-7 cells and transferrin (red) was applied for 10 min. See Materials and Methods for experimental details.

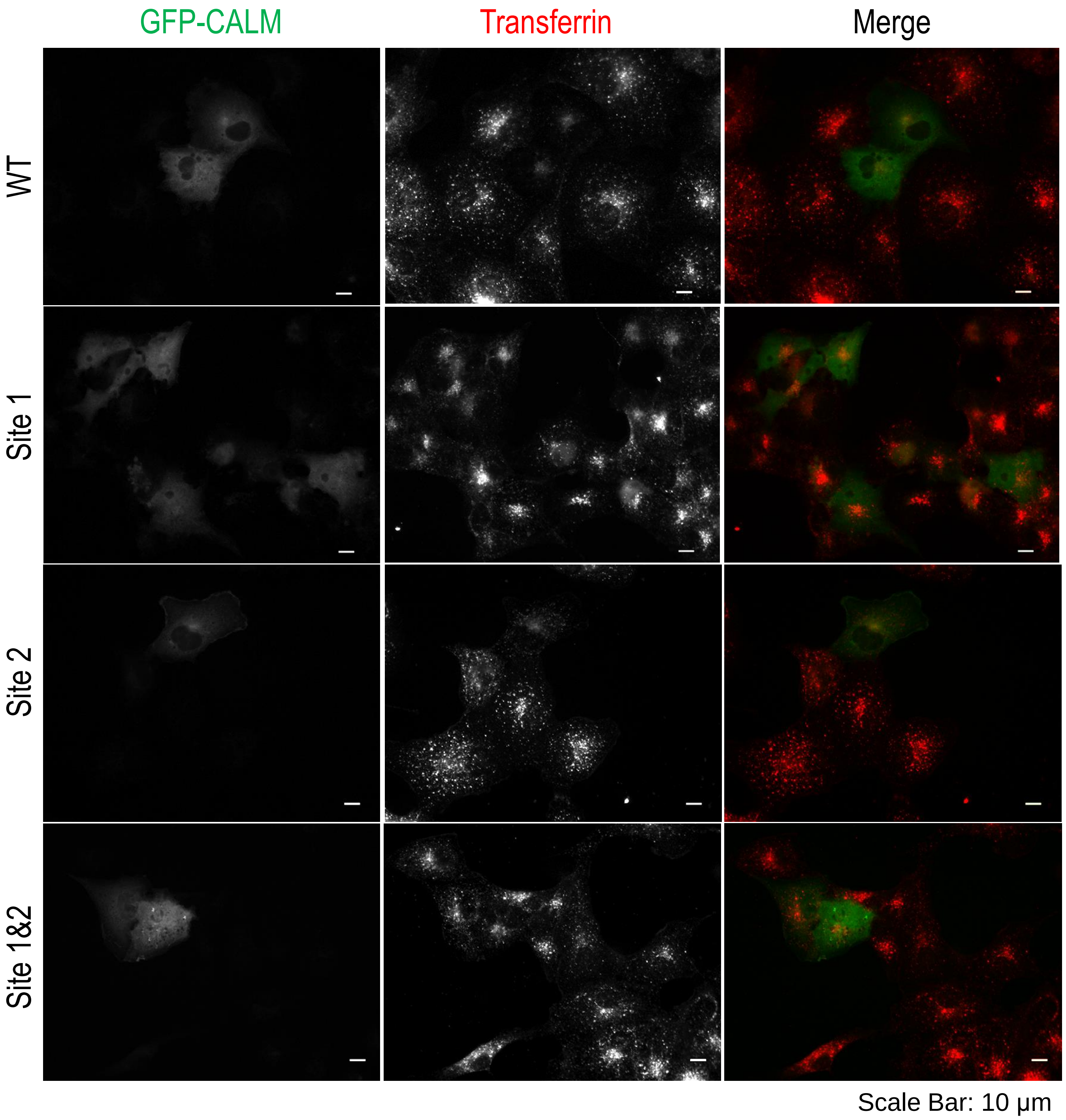

Supplement: S1 File — (PDF) [file pone.0162050.s001.pdf]
